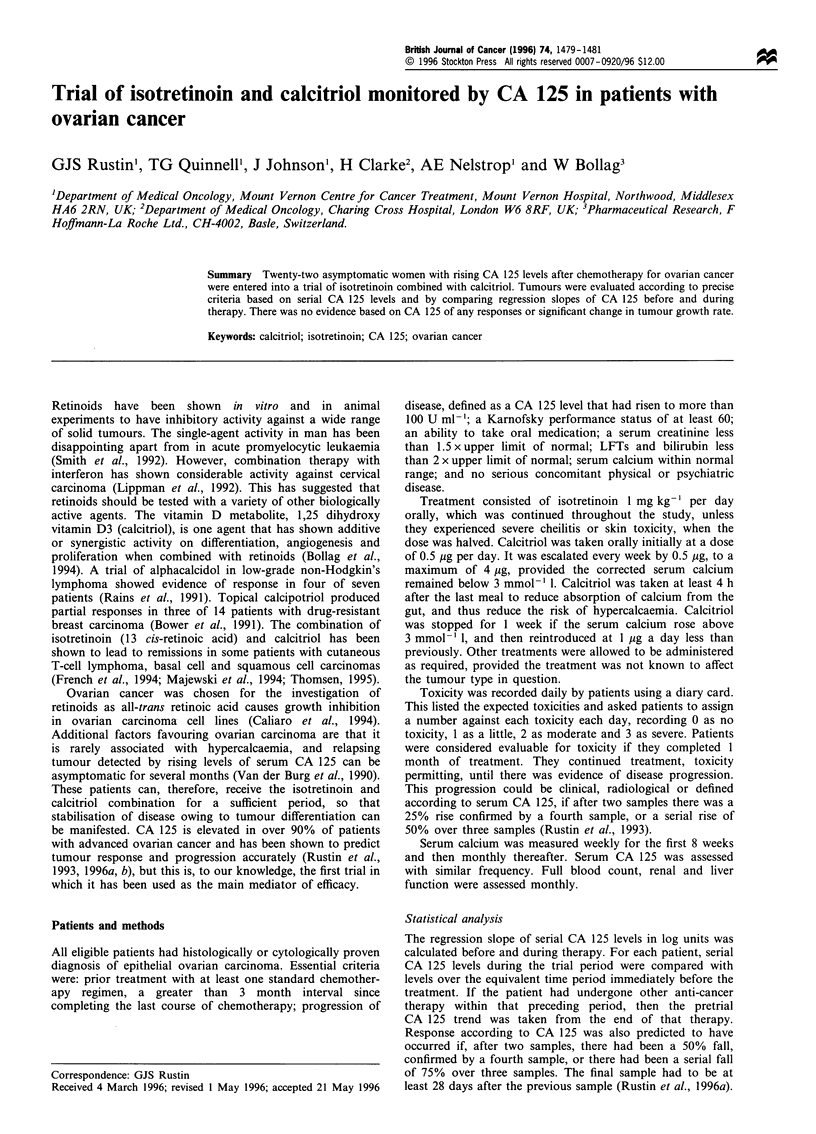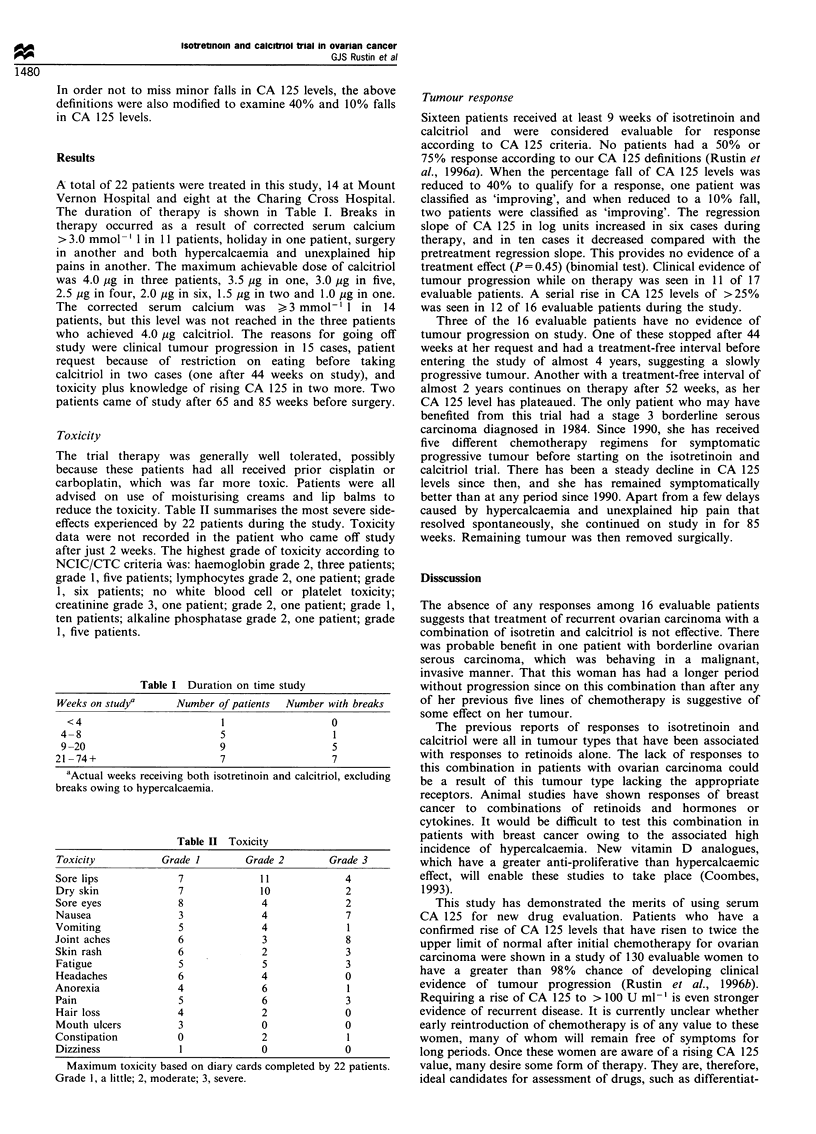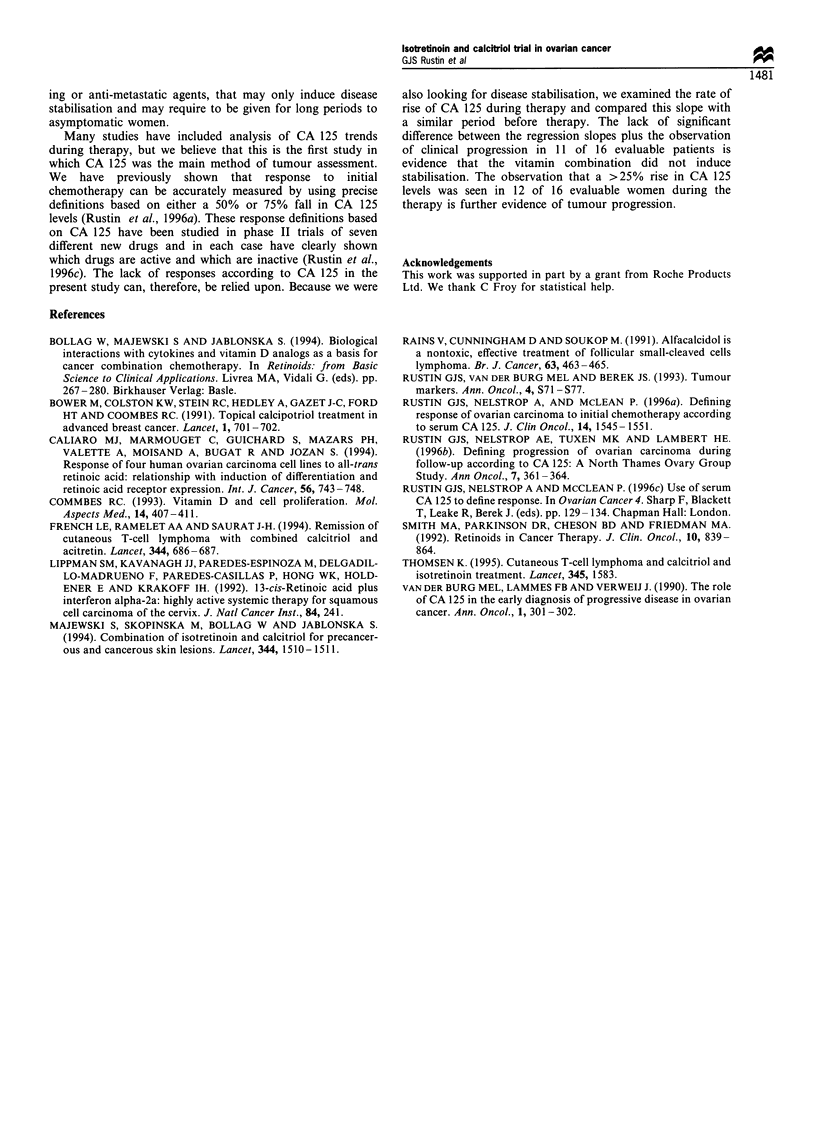# Trial of isotretinoin and calcitriol monitored by CA 125 in patients with ovarian cancer.

**DOI:** 10.1038/bjc.1996.568

**Published:** 1996-11

**Authors:** G. J. Rustin, T. G. Quinnell, J. Johnson, H. Clarke, A. E. Nelstrop, W. Bollag

**Affiliations:** Department of Medical Oncology, Mount Vernon Centre for Cancer Treatment, Mount Vernon Hospital, Northwood, Middlesex, UK.

## Abstract

Twenty-two asymptomatic women with rising CA 125 levels after chemotherapy for ovarian cancer were entered into a trial of isotretinoin combined with calcitriol. Tumours were evaluated according to precise criteria based on serial CA 125 levels and by comparing regression slopes of CA 125 before and during therapy. There was no evidence based on CA 125 of any responses or significant change in tumour growth rate.


British Journal of Cancer (1996) 74, 1479-1481
© 1996 Stockton Press All rights reserved 0007-0920/96 $12.00

### Trial of isotretinoin and calcitriol monitored by CA 125 in patients with ovarian cancer

GJS Rustin[1], TG Quinnell[1], J Johnson[1], H Clarke[2], AE Nelstrop[1] and W Bollag[3]

[1]*Department of Medical Oncology, Mount Vernon Centre for Cancer Treatment, Mount Vernon Hospital, Northwood, Middlesex HA6 2RN, UK;* [2]*Department of Medical Oncology, Charing Cross Hospital, London W6 8RF, UK;* [3]*Pharmaceutical Research, F Hoffmann-La Roche Ltd., CH-4002, Basle, Switzerland.*

**Summary** Twenty-two asymptomatic women with rising CA 125 levels after chemotherapy for ovarian cancer were entered into a trial of isotretinoin combined with calcitriol. Tumours were evaluated according to precise criteria based on serial CA 125 levels and by comparing regression slopes of CA 125 before and during therapy. There was no evidence based on CA 125 of any responses or significant change in tumour growth rate.

**Keywords:** calcitriol; isotretinoin; CA 125; ovarian cancer

Retinoids have been shown *in vitro* and in animal experiments to have inhibitory activity against a wide range of solid tumours. The single-agent activity in man has been disappointing apart from in acute promyelocytic leukaemia (Smith *et al.*, 1992). However, combination therapy with interferon has shown considerable activity against cervical carcinoma (Lippman *et al.*, 1992). This has suggested that retinoids should be tested with a variety of other biologically active agents. The vitamin D metabolite, 1,25 dihydroxy vitamin D3 (calcitriol), is one agent that has shown additive or synergistic activity on differentiation, angiogenesis and proliferation when combined with retinoids (Bollag *et al.*, 1994). A trial of alphacalcidol in low-grade non-Hodgkin's lymphoma showed evidence of response in four of seven patients (Rains *et al.*, 1991). Topical calcipotriol produced partial responses in three of 14 patients with drug-resistant breast carcinoma (Bower *et al.*, 1991). The combination of isotretinoin (13 *cis*-retinoic acid) and calcitriol has been shown to lead to remissions in some patients with cutaneous T-cell lymphoma, basal cell and squamous cell carcinomas (French *et al.*, 1994; Majewski *et al.*, 1994; Thomsen, 1995).

Ovarian cancer was chosen for the investigation of retinoids as all-*trans* retinoic acid causes growth inhibition in ovarian carcinoma cell lines (Caliaro *et al.*, 1994). Additional factors favouring ovarian carcinoma are that it is rarely associated with hypercalcaemia, and relapsing tumour detected by rising levels of serum CA 125 can be asymptomatic for several months (Van der Burg *et al.*, 1990). These patients can, therefore, receive the isotretinoin and calcitriol combination for a sufficient period, so that stabilisation of disease owing to tumour differentiation can be manifested. CA 125 is elevated in over 90% of patients with advanced ovarian cancer and has been shown to predict tumour response and progression accurately (Rustin *et al.*, 1993, 1996*a*, *b*), but this is, to our knowledge, the first trial in which it has been used as the main mediator of efficacy.

### Patients and methods

All eligible patients had histologically or cytologically proven diagnosis of epithelial ovarian carcinoma. Essential criteria were: prior treatment with at least one standard chemotherapy regimen, a greater than 3 month interval since completing the last course of chemotherapy; progression of disease, defined as a CA 125 level that had risen to more than 100 U $ml^{-1}$; a Karnofsky performance status of at least 60; an ability to take oral medication; a serum creatinine less than 1.5 × upper limit of normal; LFTs and bilirubin less than 2 × upper limit of normal; serum calcium within normal range; and no serious concomitant physical or psychiatric disease.

Treatment consisted of isotretinoin 1 mg $kg^{-1}$ per day orally, which was continued throughout the study, unless they experienced severe cheilitis or skin toxicity, when the dose was halved. Calcitriol was taken orally initially at a dose of 0.5 $\mu g$ per day. It was escalated every week by 0.5 $\mu g$, to a maximum of 4 $\mu g$, provided the corrected serum calcium remained below 3 $mmol^{-1}$ l. Calcitriol was taken at least 4 h after the last meal to reduce absorption of calcium from the gut, and thus reduce the risk of hypercalcaemia. Calcitriol was stopped for 1 week if the serum calcium rose above 3 $mmol^{-1}$ l, and then reintroduced at 1 $\mu g$ a day less than previously. Other treatments were allowed to be administered as required, provided the treatment was not known to affect the tumour type in question.

Toxicity was recorded daily by patients using a diary card. This listed the expected toxicities and asked patients to assign a number against each toxicity each day, recording 0 as no toxicity, 1 as a little, 2 as moderate and 3 as severe. Patients were considered evaluable for toxicity if they completed 1 month of treatment. They continued treatment, toxicity permitting, until there was evidence of disease progression. This progression could be clinical, radiological or defined according to serum CA 125, if after two samples there was a 25% rise confirmed by a fourth sample, or a serial rise of 50% over three samples (Rustin *et al.*, 1993).

Serum calcium was measured weekly for the first 8 weeks and then monthly thereafter. Serum CA 125 was assessed with similar frequency. Full blood count, renal and liver function were assessed monthly.

### *Statistical analysis*

The regression slope of serial CA 125 levels in log units was calculated before and during therapy. For each patient, serial CA 125 levels during the trial period were compared with levels over the equivalent time period immediately before the treatment. If the patient had undergone other anti-cancer therapy within that preceding period, then the pretrial CA 125 trend was taken from the end of that therapy. Response according to CA 125 was also predicted to have occurred if, after two samples, there had been a 50% fall, confirmed by a fourth sample, or there had been a serial fall of 75% over three samples. The final sample had to be at least 28 days after the previous sample (Rustin *et al.*, 1996*a*).

Correspondence: GJS Rustin
Received 4 March 1996; revised 1 May 1996; accepted 21 May 1996

In order not to miss minor falls in CA 125 levels, the above definitions were also modified to examine 40% and 10% falls in CA 125 levels.

### Results

A total of 22 patients were treated in this study, 14 at Mount Vernon Hospital and eight at the Charing Cross Hospital. The duration of therapy is shown in Table I. Breaks in therapy occurred as a result of corrected serum calcium $>3.0$ mmol$^{-1}$ l in 11 patients, holiday in one patient, surgery in another and both hypercalcaemia and unexplained hip pains in another. The maximum achievable dose of calcitriol was 4.0 $\mu$g in three patients, 3.5 $\mu$g in one, 3.0 $\mu$g in five, 2.5 $\mu$g in four, 2.0 $\mu$g in six, 1.5 $\mu$g in two and 1.0 $\mu$g in one. The corrected serum calcium was $\geqslant 3$ mmol$^{-1}$ l in 14 patients, but this level was not reached in the three patients who achieved 4.0 $\mu$g calcitriol. The reasons for going off study were clinical tumour progression in 15 cases, patient request because of restriction on eating before taking calcitriol in two cases (one after 44 weeks on study), and toxicity plus knowledge of rising CA 125 in two more. Two patients came of study after 65 and 85 weeks before surgery.

### *Toxicity*

The trial therapy was generally well tolerated, possibly because these patients had all received prior cisplatin or carboplatin, which was far more toxic. Patients were all advised on use of moisturising creams and lip balms to reduce the toxicity. Table II summarises the most severe side-effects experienced by 22 patients during the study. Toxicity data were not recorded in the patient who came off study after just 2 weeks. The highest grade of toxicity according to NCIC/CTC criteria was: haemoglobin grade 2, three patients; grade 1, five patients; lymphocytes grade 2, one patient; grade 1, six patients; no white blood cell or platelet toxicity; creatinine grade 3, one patient; grade 2, one patient; grade 1, ten patients; alkaline phosphatase grade 2, one patient; grade 1, five patients.

**Table I** Duration on time study

| *Weeks on study*[a] | *Number of patients* | *Number with breaks* |
|---|---|---|
| <4 | 1 | 0 |
| 4–8 | 5 | 1 |
| 9–20 | 9 | 5 |
| 21–74+ | 7 | 7 |

[a]Actual weeks receiving both isotretinoin and calcitriol, excluding breaks owing to hypercalcaemia.

**Table II** Toxicity

| *Toxicity* | *Grade 1* | *Grade 2* | *Grade 3* |
|---|---|---|---|
| Sore lips | 7 | 11 | 4 |
| Dry skin | 7 | 10 | 2 |
| Sore eyes | 8 | 4 | 2 |
| Nausea | 3 | 4 | 7 |
| Vomiting | 5 | 4 | 1 |
| Joint aches | 6 | 3 | 8 |
| Skin rash | 6 | 2 | 3 |
| Fatigue | 5 | 5 | 3 |
| Headaches | 6 | 4 | 0 |
| Anorexia | 4 | 6 | 1 |
| Pain | 5 | 6 | 3 |
| Hair loss | 4 | 2 | 0 |
| Mouth ulcers | 3 | 0 | 0 |
| Constipation | 0 | 2 | 1 |
| Dizziness | 1 | 0 | 0 |

Maximum toxicity based on diary cards completed by 22 patients. Grade 1, a little; 2, moderate; 3, severe.

### *Tumour response*

Sixteen patients received at least 9 weeks of isotretinoin and calcitriol and were considered evaluable for response according to CA 125 criteria. No patients had a 50% or 75% response according to our CA 125 definitions (Rustin *et al*., 1996*a*). When the percentage fall of CA 125 levels was reduced to 40% to qualify for a response, one patient was classified as 'improving', and when reduced to a 10% fall, two patients were classified as 'improving'. The regression slope of CA 125 in log units increased in six cases during therapy, and in ten cases it decreased compared with the pretreatment regression slope. This provides no evidence of a treatment effect ($P=0.45$) (binomial test). Clinical evidence of tumour progression while on therapy was seen in 11 of 17 evaluable patients. A serial rise in CA 125 levels of $>25\%$ was seen in 12 of 16 evaluable patients during the study.

Three of the 16 evaluable patients have no evidence of tumour progression on study. One of these stopped after 44 weeks at her request and had a treatment-free interval before entering the study of almost 4 years, suggesting a slowly progressive tumour. Another with a treatment-free interval of almost 2 years continues on therapy after 52 weeks, as her CA 125 level has plateaued. The only patient who may have benefited from this trial had a stage 3 borderline serous carcinoma diagnosed in 1984. Since 1990, she has received five different chemotherapy regimens for symptomatic progressive tumour before starting on the isotretinoin and calcitriol trial. There has been a steady decline in CA 125 levels since then, and she has remained symptomatically better than at any period since 1990. Apart from a few delays caused by hypercalcaemia and unexplained hip pain that resolved spontaneously, she continued on study in for 85 weeks. Remaining tumour was then removed surgically.

### Disscussion

The absence of any responses among 16 evaluable patients suggests that treatment of recurrent ovarian carcinoma with a combination of isotretin and calcitriol is not effective. There was probable benefit in one patient with borderline ovarian serous carcinoma, which was behaving in a malignant, invasive manner. That this woman has had a longer period without progression since on this combination than after any of her previous five lines of chemotherapy is suggestive of some effect on her tumour.

The previous reports of responses to isotretinoin and calcitriol were all in tumour types that have been associated with responses to retinoids alone. The lack of responses to this combination in patients with ovarian carcinoma could be a result of this tumour type lacking the appropriate receptors. Animal studies have shown responses of breast cancer to combinations of retinoids and hormones or cytokines. It would be difficult to test this combination in patients with breast cancer owing to the associated high incidence of hypercalcaemia. New vitamin D analogues, which have a greater anti-proliferative than hypercalcaemic effect, will enable these studies to take place (Coombes, 1993).

This study has demonstrated the merits of using serum CA 125 for new drug evaluation. Patients who have a confirmed rise of CA 125 levels that have risen to twice the upper limit of normal after initial chemotherapy for ovarian carcinoma were shown in a study of 130 evaluable women to have a greater than 98% chance of developing clinical evidence of tumour progression (Rustin *et al*., 1996*b*). Requiring a rise of CA 125 to $>100$ U ml$^{-1}$ is even stronger evidence of recurrent disease. It is currently unclear whether early reintroduction of chemotherapy is of any value to these women, many of whom will remain free of symptoms for long periods. Once these women are aware of a rising CA 125 value, many desire some form of therapy. They are, therefore, ideal candidates for assessment of drugs, such as differentiating or anti-metastatic agents, that may only induce disease stabilisation and may require to be given for long periods to asymptomatic women.

Many studies have included analysis of CA 125 trends during therapy, but we believe that this is the first study in which CA 125 was the main method of tumour assessment. We have previously shown that response to initial chemotherapy can be accurately measured by using precise definitions based on either a 50% or 75% fall in CA 125 levels (Rustin *et al.*, 1996*a*). These response definitions based on CA 125 have been studied in phase II trials of seven different new drugs and in each case have clearly shown which drugs are active and which are inactive (Rustin *et al.*, 1996*c*). The lack of responses according to CA 125 in the present study can, therefore, be relied upon. Because we were also looking for disease stabilisation, we examined the rate of rise of CA 125 during therapy and compared this slope with a similar period before therapy. The lack of significant difference between the regression slopes plus the observation of clinical progression in 11 of 16 evaluable patients is evidence that the vitamin combination did not induce stabilisation. The observation that a >25% rise in CA 125 levels was seen in 12 of 16 evaluable women during the therapy is further evidence of tumour progression.

**Acknowledgements**
This work was supported in part by a grant from Roche Products Ltd. We thank C Froy for statistical help.

### References

BOLLAG W, MAJEWSKI S AND JABLONSKA S. (1994). Biological interactions with cytokines and vitamin D analogs as a basis for cancer combination chemotherapy. In *Retinoids: from Basic Science to Clinical Applications*. Livrea MA, Vidali G. (eds). pp. 267–280. Birkhauser Verlag: Basle.

BOWER M, COLSTON KW, STEIN RC, HEDLEY A, GAZET J-C, FORD HT AND COOMBES RC. (1991). Topical calcipotriol treatment in advanced breast cancer. *Lancet*, **1**, 701–702.

CALIARO MJ, MARMOUGET C, GUICHARD S, MAZARS PH, VALETTE A, MOISAND A, BUGAT R AND JOZAN S. (1994). Response of four human ovarian carcinoma cell lines to all-*trans* retinoic acid: relationship with induction of differentiation and retinoic acid receptor expression. *Int. J. Cancer*, **56**, 743–748.

COMMBES RC. (1993). Vitamin D and cell proliferation. *Mol. Aspects Med.*, **14**, 407–411.

FRENCH LE, RAMELET AA AND SAURAT J-H. (1994). Remission of cutaneous T-cell lymphoma with combined calcitriol and acitretin. *Lancet*, **344**, 686–687.

LIPPMAN SM, KAVANAGH JJ, PAREDES-ESPINOZA M, DELGADILLO-MADRUENO F, PAREDES-CASILLAS P, HONG WK, HOLDENER E AND KRAKOFF IH. (1992). 13-*cis*-Retinoic acid plus interferon alpha-2a: highly active systemic therapy for squamous cell carcinoma of the cervix. *J. Natl Cancer Inst.*, **84**, 241.

MAJEWSKI S, SKOPINSKA M, BOLLAG W AND JABLONSKA S. (1994). Combination of isotretinoin and calcitriol for precancerous and cancerous skin lesions. *Lancet*, **344**, 1510–1511.

RAINS V, CUNNINGHAM D AND SOUKOP M. (1991). Alfacalcidol is a nontoxic, effective treatment of follicular small-cleaved cells lymphoma. *Br. J. Cancer*, **63**, 463–465.

RUSTIN GJS, VAN DER BURG MEL AND BEREK JS. (1993). Tumour markers. *Ann. Oncol.*, **4**, S71–S77.

RUSTIN GJS, NELSTROP A, AND MCLEAN P. (1996*a*). Defining response of ovarian carcinoma to initial chemotherapy according to serum CA 125. *J. Clin Oncol.*, **14**, 1545–1551.

RUSTIN GJS, NELSTROP AE, TUXEN MK AND LAMBERT HE. (1996*b*). Defining progression of ovarian carcinoma during follow-up according to CA 125: A North Thames Ovary Group Study. *Ann Oncol.*, **7**, 361–364.

RUSTIN GJS, NELSTROP A AND MCCLEAN P. (1996*c*) Use of serum CA 125 to define response. In *Ovarian Cancer 4*. Sharp F, Blackett T, Leake R, Berek J. (eds). pp. 129–134. Chapman Hall: London.

SMITH MA, PARKINSON DR, CHESON BD AND FRIEDMAN MA. (1992). Retinoids in Cancer Therapy. *J. Clin. Oncol.*, **10**, 839–864.

THOMSEN K. (1995). Cutaneous T-cell lymphoma and calcitriol and isotretinoin treatment. *Lancet*, **345**, 1583.

VAN DER BURG MEL, LAMMES FB AND VERWEIJ J. (1990). The role of CA 125 in the early diagnosis of progressive disease in ovarian cancer. *Ann. Oncol.*, **1**, 301–302.